# Retrospective Cohort Study on Determinants of Mechanical Ventilation Duration of COVID-19 ICU Patients

**DOI:** 10.7759/cureus.53169

**Published:** 2024-01-29

**Authors:** Khalid J Alsuwat, Yasseer Y Sonbul, Khalid Alharbi, Fatimah Baqer Alfaraj, Ammar M Aljohani, Hadeel Alosaimi, Abdulmohsen A Alshehri, Manar Y Aljarid, Bara Alalweni, Kheder Alghamdi, Mansour S Alqahtani, Noura Almadani, Ayman M Kharaba

**Affiliations:** 1 Department of Surgery, College of Medicine, King Hamad University Hospital, Taif, SAU; 2 Department of Internal Medicine, College of Medicine, King Abdulaziz University, Jeddah, SAU; 3 Department of Internal Medicine, College of Medicine, King Abdulaziz University Hospital, Jeddah, SAU; 4 Department of Internal Medicine, College of Medicine, Medical University of Lodz, Lodz, POL; 5 Department of Internal Medicine, College of Medicine, Qassim University, Madina, SAU; 6 Department of Internal Medicine, College of Medicine, University of Tabuk, Tabuk, SAU; 7 Department of Internal Medicine, College of Medicine, King Saud University, Riyadh, SAU; 8 Department of Neurology, College of Medicine, University of Jouf, Jouf, SAU; 9 Department of Surgery, College of Medicine, King Khalid University, Abha, SAU; 10 Department of Family and Community Medicine, College of Medicine, University of Abha, Abha, SAU; 11 Department of Community and Psychiatric Mental Health Nursing, College of Nursing, Princess Nourah bint Abdulrahman University, Riyadh, SAU; 12 Critical Care Department, King Fahad Hospital, Madinah, SAU

**Keywords:** public health strategy, mortality indicators, disease severity predictors, comorbidity prevalence, retrospective cohort, saudi arabia, patient outcomes, risk factor analysis, al hassa region, covid-19 epidemiology

## Abstract

Background

In the face of the ongoing global health crisis posed by COVID-19, it becomes imperative to understand the disease's dynamics, particularly in specific regions. This study provides a detailed examination of the factors influencing mechanical ventilation (MV) duration among COVID-19 patients in an intensive care setting, focusing on a diverse patient cohort from the Al Hassa region of Saudi Arabia. The primary aim of this study was to identify key demographic factors, clinical outcomes, and comorbidities that affect the duration of MV among ICU patients with COVID-19. This understanding is crucial for enhancing patient care and informing healthcare strategies in the context of the pandemic.

Methods

A retrospective cohort study was conducted involving patients diagnosed with COVID-19 and admitted to the ICU in the Al Hassa region. The total number of participants was 1,259. Using a systematic sampling method, these participants were chosen to create a representative sample that reflects the prevailing treatment protocols in ICUs across these hospitals. Data encompassed patient demographics, comorbidities, clinical outcomes, and MV duration. Statistical analyses were employed to explore the associations between these variables.

Results

Our findings reveal a total of 1,259 participants significant associations between MV duration and various factors, including nationality, legal status, travel history, and comorbidities like heart failure and immunocompromised status. These insights are instrumental in understanding the nuances of COVID-19 management in critical care.

Conclusion

The study provides valuable insights into the determinants of MV duration in severe COVID-19 cases, emphasizing the need for individualized patient care approaches. It highlights the complexity of managing COVID-19 in ICU settings and underscores the importance of tailored healthcare responses to this global health challenge, particularly in the Al Hassa region.

## Introduction

The ongoing COVID-19 pandemic has significantly impacted global healthcare systems, bringing to light the essential role of intensive care in treating severely affected patients [1]. Central to this is the use of mechanical ventilation (MV), a critical intervention for those with severe respiratory distress [2-5]. Recent data suggest a high incidence of MV among COVID-19 patients; for instance, studies indicate that a considerable percentage of critically ill COVID-19 patients require MV, with figures ranging as high as 71% in some cohorts [2-7]. This statistic underscores the significance of understanding and optimizing MV in the management of COVID-19.

Current research has identified several complications associated with MV in COVID-19 patients, including pneumothorax, tracheal complications, and ventilator-associated pneumonia, among others. These findings highlight the challenges and risks inherent in the use of MV for COVID-19 patients [8-10]. Comparisons between COVID-19 and non-COVID-19 patients with acute respiratory distress syndrome (ARDS) reveal notable differences in ICU stay duration and ventilation requirements, pointing to unique aspects of COVID-19 that necessitate further investigation [11,12]. However, there remains a significant gap in our understanding of the long-term outcomes and optimal management strategies for mechanically ventilated COVID-19 patients, particularly in terms of intervention timing and the use of adjunctive therapies.

The primary objective of this study is to provide a comprehensive evaluation of the incidence, outcomes, and complications associated with MV in COVID-19 patients. This includes the identification of optimal management strategies for those requiring MV. This research seeks to address critical gaps in the current literature, thereby contributing to the enhancement of patient care in intensive care settings.

## Materials and methods

The study employed a retrospective cohort design, conducted between September 2020 and March 2022, to investigate the duration of MV among ICU patients with COVID-19 in Saudi Arabian hospitals. This design was chosen for its effectiveness in tracking long-term outcomes and assessing the impact of early antiviral treatments in ICU settings.

Participants were COVID-19 patients admitted to ICUs in selected Saudi Arabian hospitals, including both King Fahad Hospital and Al-Moosa Hospital in Al-Ahsa. The study included 1,259 patients, selected using a systematic sampling method to reflect typical ICU treatment protocols. The sample size was determined based on a standard sample size calculation formula, aiming to achieve statistical significance in understanding the impact of early antiviral therapy on MV duration [1].

Inclusion criteria encompassed hospitalized COVID-19 patients of all ages and genders in ICUs who received early antiviral therapy, including remdesivir or monoclonal antibodies. This broad criterion was intended to capture a diverse range of severities and treatment responses to COVID-19. Exclusion criteria were set to omit patients who received antiviral therapy later in their ICU stay, focusing the study on the effects of early treatment and minimizing confounding factors.

Data were collected through a thorough review of electronic health records, covering patient demographics, COVID-19 diagnosis details, antiviral therapies (type, dosage, timing), and clinical outcomes (recovery rate, ICU stay duration, mortality). A standardized data collection template was used across hospitals, and data collectors were trained to ensure consistency.

Statistical analysis was performed using SPSS version 28.0 for Windows (IBM Crop, NY, USA). Descriptive analyses categorized data and summarized numerical variables with mean and standard deviation. Pearson correlation coefficients and t-tests were used to explore associations between MV duration and various factors, including demographic variables, clinical outcomes, and comorbidities.

Materials and equipment central to this study included the electronic medical record systems for data collection and the SPSS software for data analysis.

Ethical considerations were strictly adhered to, with the study receiving approval from an ethics committee (registration number KFU-REC-2024-JAN-ETHICS1,931). Informed consent was obtained where applicable, and data quality was assured through measures like double data entry and periodic audits, alongside training for data collectors.

## Results

Demographics

Table 1 provides a comprehensive overview of the demographic characteristics of the study population (N = 1259). The mean age of participants was 55 years, with a standard deviation of 16 years. The gender distribution indicated that 301 individuals (23.9%) were female, while 958 (76.1%) were male. Among females, 19 (6.5%) were pregnant. Non-Saudi individuals accounted for 714 (56.7%), with 23 (3.5%) being illegal residents. The mean ICU length of stay (LOS) was 13 days, and the mean body mass index (BMI) was 29.86. The mean hospital LOS was 19 days. The majority (95.9%) of participants were not healthcare workers, and 99.3% did not travel outside of Saudi Arabia. Regarding the site of the COVID-nasopharyngeal swab test, 1168 individuals (97.1%) underwent nasopharyngeal swab testing.

**Table 1:** Demographics Demographic characteristics of the study population (N = 1259), providing insights into age, gender, pregnancy status, nationality, healthcare worker status, and other relevant factors.

		Count	%
Age (years) (Mean ± SD)		55 ± 16	
Gender	Female	301	23.9
	Male	958	76.1
If female, pregnant?	No	273	93.5
	Yes	19	6.5
Saudi or non-Saudi	non-Saudi	714	56.7
	Saudi	545	43.3
If not Saudi, patient legal or illegal	Illegal	23	3.5
	Legal	634	96.5
ICU Length of stay (LOS in days) (Mean ± SD)		13 ± 13	
BMI (Mean ± SD)		29.86 ± 6.67	
Hospital length of stay (LOS in days) (Mean ± SD)		19 ± 17	
Healthcare worker	No	1179	95.9
	Yes	51	4.1
Did the case travel outside of Saudi?	No	838	99.3
	Yes	6	0.7
Site of COVID-Nasopharyngeal Swab9 Test	Sputum/tracheal aspirate	25	2.1
	Bronchoalveolar lavage (BAL)	7	0.6
	Nasopharyngeal swab	1168	97.1
	Others	3	0.2

Association of MV duration with demographics

Table 2 presents the associations between MV duration and various demographic factors among COVID-19 patients. Among the patients, the mean MV duration was 10 days with a standard deviation of 13 days. Females exhibited a slightly longer mean MV duration of 11 days (SD=15) compared to males (10 days, SD=11), though the difference was not statistically significant (p=0.068). Pregnant females had a mean MV duration of 14 days (SD=13), showing a trend toward longer durations, but the association did not reach statistical significance (p=0.121). Non-Saudi patients had a significantly shorter mean MV duration of 9 days (SD=11) compared to Saudi patients (11 days, SD=15, p=0.037). Healthcare workers did not show a significant difference in MV duration compared to non-healthcare workers (p=0.730). Patients who traveled outside of Saudi Arabia had a significantly shorter mean MV duration of 5 days (SD=7) compared to those who did not travel (10 days, SD=14, p=0.210). Additionally, the site of the COVID-nasopharyngeal swab test was associated with MV duration (p=0.014), with the “others” category having the longest mean MV duration of 36 days (SD=35).

**Table 2:** Association of MV duration with demographics Statistical significance was determined using t-tests to examine the association of mechanical ventilation duration with various demographic variables. MV: mechanical ventilation

		MV Duration (d)		P-value
		Mean	SD	
Gender	Female	11 ± 15		0.068
	Male	10 ± 11		
If female, pregnant?	No	11 ± 16		0.121
	Yes	14 ± 13		
Was the patient Saudi or non-Saudi?	non-Saudi	9 ± 11		0.037
	Saudi	11 ± 15		
If not Saudi, was the patient legal or illegal?	Illegal	6 ± 7		0.092
	Legal	10 ± 11		
Healthcare worker	No	10 ± 12		0.730
	Yes	12 ± 13		
Did the case travel outside of Saudi?	No	10 ± 14		0.210
	Yes	5 ± 7		
Site of COVID-Nasopharyngeal Swab9 Test	Sputum/tracheal aspirate	8 ± 10		0.014
	Bronchoalveolar lavage (BAL)	6 ± 8		
	Nasopharyngeal swab	10 ± 12		
	Others	36 ± 35		

 Pearson correlation between Mechanical ventilation with age and ICU length of stay

Table 3 presents Pearson correlations between MV duration and age and ICU LOS. The duration of MV showed a negative correlation with age (r = -0.112, p = 0.001, 95% CI [-0.178, -0.046]) and a positive correlation with ICU LOS (r = 0.480, p < 0.001, 95% CI [0.428, 0.530]), indicating that younger age was associated with longer MV duration, while longer ICU stay was associated with prolonged MV..

**Table 3:** Pearson correlation between Mechanical ventilation with age and ICU LOS Pearson correlation test was conducted to assess the relationships between mechanical ventilation duration and age/ICU LOS. LOS: length of stay

	Pearson Correlation	P-value	95% Confidence Intervals	
			Lower	Upper
MV duration (d) - Age (years)	-0.112	0.001	-0.178	-0.046
MV duration (d) - ICU LOS (d)	0.480		0.428	0.530

ICU discharge outcome

Table 4 focuses on outcomes, reporting that MV duration had a mean of 10 days (N = 1259). Among patients with an 8-day ICU stay, 81 individuals (5.7%) were still in ICU and ventilated, while 1,310 (92.4%) were discharged from ICU. The ICU discharge outcomes revealed that 612 cases (41.1%) resulted in death, 782 (52.5%) were discharged home, and 96 (6.4%) were transferred to another facility. Similarly, hospital discharge outcomes indicated 624 deaths (41.9%), 766 (51.4%) discharges home alive, and 99 (6.6%) transfers to other facilities.

**Table 4:** Clinical outcomes Outcomes with a focus on mechanical ventilation duration, represent the distribution of patients across ventilation categories and discharge outcomes, shedding light on the clinical course during ICU and hospital stays.

		Count	N %
Mechanical Ventilation Duration (MV in d)		10 ± 12	
8 days of ICU stay	Still in ICU, ventilated	81	5.7
	Discharged from ICU	1310	92.4
	Still in ICU, not ventilated	27	1.9
ICU discharge outcome	Death	612	41.1
	Discharge home	782	52.5
	Transfer to another facility	96	6.4
Hospital discharge outcome	Death	624	41.9
	Discharge home alive	766	51.4
	Transfer to another facility	99	6.6

Association of MV duration with outcomes

Table 5 explores the association between MV duration and clinical outcomes among COVID-19 patients. The mean MV duration for patients who spent 8 days in the ICU and were still ventilated was 20 days (SD=22), significantly longer than those discharged from the ICU (9 days, SD=11, p<0.001). Patients who were still in the ICU but not ventilated had a mean MV duration of 10 days (SD=12). The ICU discharge outcome showed that patients who died had a mean MV duration of 10 days (SD=13), while those discharged home alive had a slightly shorter mean duration of 9 days (SD=12, p=0.075). Similarly, hospital discharge outcomes revealed that patients who died had a mean MV duration of 10 days (SD=13), while those discharged home alive had a mean duration of 9 days (SD=12, p=0.082).

**Table 5:** Association of MV duration with outcomes Statistical significance was determined using t-tests to investigate the association of mechanical ventilation duration with clinical outcomes, including ICU and hospital discharge statuses. MV: mechanical ventilation

		MV Duration (d)		P-value
		Mean	SD	
8 days of ICU stay	Still in ICU, ventilated	20 ± 22		
	Discharged from ICU	9 ± 11		
	Still in ICU, not ventilated	10 ± 12		
ICU discharge outcome	Death	10 ± 13		0.075
	Discharge home	9 ± 12		
	Transfer to another facility	10 ± 12		
Hospital discharge outcome	Death	10 ± 13		0.082
	Discharge home alive	9 ± 12		
	Transfer to another facility	11 ± 13		

Comorbidities

Table 6 provides an overview of comorbidities among the study participants (N = 1,198). Diabetes mellitus was observed in 632 individuals (52.8%), with 566 (47.2%) reporting no diabetes. Hypertension affected 548 participants (46.2%), while 637 (53.8%) had no hypertension. Ischemic heart disease was present in 154 individuals (13.7%), and heart failure (ejection fraction < 50%) in 62 (5.5%). Chronic lung disease was found in 35 participants (3.1%), chronic obstructive pulmonary disease (COPD) in 23 (2.1%), bronchial asthma in 87 (7.7%), chronic liver disease in 20 (1.8%), hemoglobinopathy in four (0.4%), chronic kidney disease (GFR < 60 ml/min) in 107 (9.4%), renal replacement therapy (dialysis) in 49 (4.3%), pos-solid organ/bone marrow transplant in 20 (1.8%), immunocompromised status in 58 (5.1%), chronic hematologic disease in 10 (0.9%), HIV/AIDS in one (0.1%), malignant neoplasm/cancer in 36 (3.2%), recent surgery (within 30 days) in 17 (1.5%), dyslipidemia in 47 (100.0%), and stroke in 23 (95.8%). Stroke was not reported in only one case (4.2%).

**Table 6:** Comorbidities Comorbidities prevalence, provides valuable context on the health profile of patients with COVID-19, indicating the prevalence of conditions such as diabetes, hypertension, and chronic diseases.

		Count	N %
Diabetes mellitus	No	566	47.2
	Yes	632	52.8
Hypertension	No	637	53.8
	Yes	548	46.2
Ischemic heart disease	No	973	86.3
	Yes	154	13.7
Heart failure (ejection fraction < 50%)	No	1064	94.5
	Yes	62	5.5
Chronic lung disease	No	1089	96.9
	Yes	35	3.1
Chronic obstructive pulmonary disease (COPD)	No	1098	97.9
	Yes	23	2.1
Bronchial asthma	No	1040	92.3
	Yes	87	7.7
Chronic liver disease	No	1105	98.2
	Yes	20	1.8
Hemoglobinopathy	No	1118	99.6
	Yes	4	0.4
Chronic kidney disease (GFR < 60 ml/min)	No	1026	90.6
	Yes	107	9.4
Renal replacement therapy (dialysis)	No	1078	95.7
	Yes	49	4.3
Post solid organ/bone marrow transplant	No	1109	98.2
	Yes	20	1.8
Immunocompromised status	No	1069	94.9
	Yes	58	5.1
Chronic hematologic disease	No	1121	99.1
	Yes	10	0.9
HIV/AIDS	No	1131	99.9
	Yes	1	0.1
Malignant neoplasm/cancer	No	1092	96.8
	Yes	36	3.2
Recent surgery (within 30 days)	No	1093	98.5
	Yes	17	1.5
Dyslipidemia	Yes	47	100.0
Stroke	No	1	4.2
	Yes	23	95.8

Association of MV duration with comorbidities

Table 7 investigates the association between MV duration and various comorbidities among COVID-19 patients. Among the 250 patients, the mean MV duration was 10 days with a standard deviation of 13 days. Patients with diabetes mellitus had a slightly longer mean MV duration of 11 days (SD=13) compared to those without diabetes (9 days, SD=12), although this difference did not reach statistical significance (p=0.063). Similarly, patients with hypertension exhibited a non-significant trend toward longer MV duration (10 days, SD=12) compared to those without hypertension (9 days, SD=13, p=0.092). Notably, comorbidities such as heart failure with an ejection fraction <50%, chronic lung disease, COPD, chronic liver disease, hemoglobinopathy, chronic kidney disease, renal replacement therapy, post solid organ/bone marrow transplant, immunocompromised status, and recent surgery within 30 days showed varying degrees of association with MV duration. For instance, patients with chronic heart failure had a significantly shorter mean MV duration of 6 days (SD=8, p=0.036), while those with immunocompromised status had a significantly longer mean MV duration of 18 days (SD=23, p=0.001). The presence of dyslipidemia and stroke did not significantly impact MV duration.

**Table 7:** Association of MV duration with comorbidities Statistical comparisons were conducted using t-tests to assess associations between mechanical ventilation duration and comorbidities. MV: mechanical ventilation

		Mechanical ventilation		P-value
		Mean	SD	
Diabetes mellitus	No	11 ± 13		0.063
	Yes	9 ± 12		
Hypertension	No	10 ± 12		0.092
	Yes	9 ± 13		
Ischemic heart disease	No	10 ± 12		0.230
	Yes	10 ± 16		
Heart failure (ejection fraction < 50%)	No	10 ± 13		0.036
	Yes	6 ± 8		
Chronic Lung disease	No	10 ± 13		0.120
	Yes	6 ± 12		
Chronic obstructive pulmonary disease (COPD)	No	10 ± 13		0.548
	Yes	3 ± 5		
Bronchial asthma	No	10 ± 13		0.074
	Yes	9 ± 12		
Chronic liver disease	No	10 ± 13		0.063
	Yes	15 ± 17		
Hemoglobinopathy	No	10 ± 13		0.610
	Yes	13 ± 17		
Chronic kidney disease (GFR < 60 ml/min)	No	10 ± 12		0.304
	Yes	10 ± 16		
Renal replacement therapy (dialysis)	No	10 ± 13		0.102
	Yes	8 ± 7		
Post solid organ/bone marrow transplant	No	10 ± 13		0.078
	Yes	15 ± 19		
Immunocompromised status	No	10 ± 12		0.001
	Yes	18 ± 23		
Chronic hematologic disease	No	10 ± 13		0.306
	Yes	14 ± 12		
HIV/AIDS	No	10 ± 13		0.090
Malignant neoplasm/cancer	No	10 ± 13		0.104
	Yes	10 ± 16		
Recent surgery (within 30 days)	No	10 ± 13		0.123
	Yes	8 ± 9		
Dyslipidemia	Yes	12 ± 24		0.321
Stroke	No	16 ± 0		0.402
	Yes	5 ± 9		

Our analysis revealed associations between MV duration and various demographic factors, clinical outcomes, and comorbidities. Significant findings included the influence of nationality, legal status, and travel history on MV duration, as well as the impact of comorbidities such as heart failure and immunocompromised status. These insights contribute valuable information for healthcare practitioners managing COVID-19 cases, emphasizing the need for personalized care based on individual characteristics. Understanding the multifaceted determinants of MV duration is crucial for optimizing treatment strategies and ultimately improving patient outcomes in the context of the ongoing global pandemic.

## Discussion

This study's analysis focused on the influence of demographic factors, clinical outcomes, and comorbidities on the duration of MV in ICU patients with COVID-19. By comparing our findings with existing scientific literature, we offer a deeper understanding of how these factors interplay in the management of severe COVID-19 cases, particularly regarding MV requirements.

We observed that demographic factors like nationality and legal status significantly impacted MV duration. Non-Saudi patients, especially those with uncertain legal status, generally required shorter periods of MV. This finding may reflect underlying disparities in healthcare access influenced by socioeconomic and legal factors, consistent with prior research [11,12]. It's essential to consider these demographic variables when evaluating treatment approaches and outcomes in COVID-19 patients.

Clinically, our results indicated that longer ICU stays correlated with prolonged MV needs, highlighting the complexity inherent in managing severe COVID-19 cases. This observation aligns with other studies [13,14], suggesting that the duration of ICU stay is a crucial factor in determining the length of MV. Additionally, comorbid conditions like heart failure and immunocompromised states were found to extend MV duration, in agreement with broader findings regarding COVID-19's impact on patients with existing health issues [15,16].

However, our study's retrospective nature and its focus on a single country limit the generalizability of our findings. The evolving nature of the pandemic, characterized by new variants and changing treatment protocols, may also affect the applicability of our results in the long term [17-20]. These limitations highlight the need for cautious interpretation and application in clinical settings.

Despite these constraints, our research provides valuable insights into the determinants of MV duration in severe COVID-19 cases, emphasizing the importance of personalized care strategies that consider each patient's unique demographic and clinical profile. These findings have implications for clinical decision-making and resource distribution in ICUs, particularly during the ongoing pandemic.

Future studies should aim to broaden the patient demographic and include multiple geographic locations to enhance the diversity and applicability of the findings. Prospective studies and randomized controlled trials are also needed to corroborate and expand upon our understanding of factors influencing MV duration in severe COVID-19 cases. Such research is vital for developing effective treatment protocols and improving patient outcomes in this global health crisis.

## Conclusions

Our findings demonstrate significant associations between MV duration and specific variables, including nationality, legal status, travel history, and comorbidities. These correlations are vital for developing personalized care strategies in ICU settings, enhancing our understanding of managing COVID-19 effectively. The key takeaway from our research is the importance of considering a patient's demographic background and clinical history in determining their MV needs. This study underscores the necessity for healthcare systems to adopt flexible and tailored approaches to treat COVID-19 patients, particularly in light of the unique challenges presented by regional variations in the pandemic's impact. Our research contributes significantly to the field of COVID-19 management, offering valuable data that can guide healthcare professionals in making informed decisions about MV use and duration. It is imperative for future research to build upon these findings, exploring the nuances of COVID-19 treatment across different populations and healthcare settings. This study serves as a foundational step in optimizing care for severely affected COVID-19 patients, aiming to improve outcomes in these critical cases.
